# EFFECT OF SEMAGLUTIDE ON PHYSICAL FUNCTION, BODY COMPOSITION, AND BIOMARKERS OF AGING IN OLDER ADULTS

**DOI:** 10.1093/geroni/igae098.1054

**Published:** 2024-12-31

**Authors:** Tiffany Cortes, Ronna Robbins, Monica Serra, Sara Espinoza, Nicolas Musi, Hannah Barrus, Kevin Brown, Annalis Campos

**Affiliations:** The University of Texas Health San Antonio, San Antonio, Texas, United States; Texas Woman’s University, Denton, Texas, United States; The University of Texas Health San Antonio, San Antonio, Texas, United States; Cedars-Sinai Medical Center, Los Angeles, California, United States; Cedars-Sinai Medical Center, Los Angeles, California, United States; The University of Texas Health San Antonio, San Antonio, Texas, United States; The University of Texas Health San Antonio, San Antonio, Texas, United States; The University of Texas Health San Antonio, San Antonio, Texas, United States

## Abstract

Insulin resistance and obesity are associated with sarcopenia and functional decline, but these important outcomes are not usually considered when choosing medications for diabetes or body weight management. Semaglutide is a GLP1 receptor agonist that typically results in average reductions in hemoglobin A1c of 1.5% and weight loss of 12%. The aims of this project are to (1) determine if semaglutide can improve physical function and muscle mass in older adults with prediabetes or diabetes and (2) elucidate whether these changes are associated with changes in inflammatory and age-related biomarkers in blood, adipose, and skeletal muscle. To accomplish these aims, we are conducting a 20-week open label, randomized trial with older adults with prediabetes or diabetes and who are overweight or obese. As of March 2024, we have enrolled 20 older adults, 50% women, with an average (standard deviation) age of 76.7 (4.7) years, BMI 32.9 (3.9) kg/m2, and hemoglobin A1c 6.0 (0.4) %. Participants are randomized 1:1 to receive either semaglutide 1mg plus lifestyle counseling or lifestyle counseling only for 20 weeks. Measures of physical function (6 minute walk, short physical performance battery, and grip strength), body composition (lean and fat mass via DXA), and aging biomarkers (IL6, TNFα, and CRP) are obtained at baseline and at end of study. We hypothesize that this study will provide insight on the role of GLP1 receptor agonists in overweight and obese adults with prediabetes or diabetes on important clinical outcomes beyond glycemic control. This study will be completed May 2024.

